# Fish odor syndrome (trimethylaminuria) supporting the possible *FMO3* down expression in childhood: a case report

**DOI:** 10.1186/1752-1947-8-328

**Published:** 2014-10-06

**Authors:** Rosalia D’Angelo, Concetta Scimone, Teresa Esposito, Daniele Bruschetta, Carmela Rinaldi, Alessia Ruggeri, Antonina Sidoti

**Affiliations:** 1 Department of Biomedical Sciences and Morphological and Functional Images, Division of Medical Biotechnologies and Preventive Medicine, University of Messina, via C. Valeria 1, I-98125 Messina, Italy; 2 IRCCS Centro Neurolesi “Bonino-Pulejo”, Messina, Italy; 3 Department of Experimental Medicine, Sec. Bottazzi, Second University of Naples, Naples, Italy

**Keywords:** Fish odor syndrome, Flavin-containing monooxygenase, *FMO3*, Trimethylamine, Trimethylamine *N*-oxide, Trimethylaminuria

## Abstract

**Introduction:**

Trimethylaminuria is a rare inherited disorder due to decreased metabolism of dietary-derived trimethylamine by flavin-containing monooxygenase 3. Several single nucleotide polymorphisms of the flavin-containing monooxygenase 3 gene have been described and result in an enzyme with decreased or abolished functional activity for trimethylamine *N*-oxygenation thus leading to trimethylaminuria.

**Case presentation:**

Here we investigated an Italian family in which the proband was a 7-year-old girl with suspected trimethylaminuria, by flavin-containing monooxygenase 3 gene direct sequencing and urinary determination of trimethylamine and trimethylamine *N*-oxide. Genetic analysis found that, as with her parents and one of her two brothers, the proband carried three polymorphisms: c.472 G>A p. E158K (rs 2266782) in exon 4, c.627+10 C>G (IVS5+10G>C) (rs 2066534) and c.485-21 G>A (IVS4-22G>A) (rs 1920149) in intronic regions.

**Conclusions:**

Despite the same genotypic condition only the girl had symptoms attributable to the trimethylaminuria. The suspicion is that she has transient childhood trimethylaminuria. Therefore, we bring attention to the importance of genetic testing and eventual determination of urinary trimethylamine and trimethylamine *N*-oxide as instruments to offer to clinicians in the management of these pediatric patients.

## Introduction

Flavin-containing monooxygenase 3 (*FMO3*) is one of five members of a flavin-containing monooxygenases (*FMOs*) gene family whose gene products are localized in the endoplasmic reticulum of many tissues where they catalyze the nicotinamide adenine dinucleotide phosphate -dependent oxidative metabolism of many drugs, pesticides, dietary components and other foreign compounds.

FMO3 is the major adult hepatic isoform whereas FMO1 is the major fetal isoform. Both are subject to developmental and tissue-specific regulation, with a developmental switch in the expression of *FMO1* and *FMO3* genes taking place in the liver [1,2]. So, FMO1 is the childhood form and FMO3 is expressed in late childhood and into adulthood. *FMO3* is responsible for *N*-oxidation of a malodorous metabolite, trimethylamine (TMA), to the non-odorous trimethylamine *N*-oxide (TMAO); it is present in low abundance in fetal liver and it is expressed at intermediate levels until 11 years of age with an increase in its expression during puberty. TMA is derived from dietary precursors, such as choline and lecithin, found in foods such as egg yolk, liver, kidney, legumes, soybeans, peas, shellfish, and salt-water fish via the action of bacteria in the human gut.

More than 300 single nucleotide polymorphisms of the human *FMO3* have been reported [3] and over 40 of these polymorphisms have been linked to trimethylaminuria (TMAU) also known as “fish odor syndrome”. Affected individuals excrete excessive amounts of TMA in sweat, saliva, urine, breath, and vaginal secretions. TMAU is not associated with mortality or morbidity, but psychosocial consequences may be devastating. Two major forms of TMAU have been described [4]: a primary genetic form that causes decreased FMO3 function, and a secondary one that is due to TMA or to a TMA-precursor overload. It is an autosomal recessive disorder, but there are minor forms of TMAU including an acquired TMAU with no obvious *FMO3* background, a transient childhood form, and a transient form in women associated with menstruation [5-7].

In this study we report a case of suspected TMAU in a 7-year-old girl.

## Case presentation

An Italian 7-year-old girl with a TMAU-like phenotype has come to our attention after her mother reported the production of strong body odor. The child’s history revealed that she is the third child of healthy, unrelated parents. Her two brothers aged 16 and 10 years were both apparently healthy.

All her hematological parameters and her biochemical indices for renal, thyroid and liver function were within the normal range.

TMAU was suspected and it was suggested that the child’s DNA be examined for mutations in the *FMO3* gene. The study was approved by our local ethics committee. Written informed consent was obtained from the patient’s parents.

Molecular analysis of *FMO3* gene in the index patient and family members was performed. Genomic DNA was extracted from heparinized peripheral blood of all family members using the salting out method [8]. Upstream sequence, the non-coding exon 1 and each of the coding exons (exons 2 to 9) of the *FMO3* gene were amplified from genomic DNA by polymerase chain reaction using the primer pairs shown in Table 1.

**Table 1:** Primer pairs employed in polymerase chain reaction analysis

**Primer set**	**Primer sequence**	Ta*	**Target**
1	5'-ACCACACACTGTACTCAGA-3'	51°C	Upstream
	5'-GTCTACGTCCTGTCCAAC-3'		
2	5'-GTAGGAGGCTGAGGCGGG-3'	52°C	Exon1
	5'-GTTTCACCATGTTGGTCA-3'		
3	5'-GTGAGCTACCATACTCA-3'	52°C	Exon2
	5'-CTGTGTGCACACAGTGT-3'		
4	5'-GATGACTGTAATTACTTGG-3'	52°C	Exon3
	5'-ATGAGAATCCAGTA-3'		
5	5'-TGTAATATGTATCTTAATCAT-3'	43°C	Exon4
	5'-TGTCAGTTATGTGGCTA-3'		
6	5'-CATTATTGTGACTGCATC-3'	50°C	Exon5
	5'-AACTCTTCTGTCAGTAAC-3'		
7	5'-GTAATAGATCCATTCCTCA-3'	50°C	Exon6
	5'-GCTTACAGGACATTAAG-3'		
8	5'-TATATATGGACCAATAAAAC-3'	50°C	Exon7
	5'-GTCACTGGCATTCATCT-3'		
9	5'ACACCAATTAATGTAATTCA-3'	52°C	Exon8
	5'-TCTATGGAAATCCTACAC-3'		
10	5'-TCTGTTCTGTTTCTACAC-3'	52°C	Exon9
	5'-GATGATTAGGTCAACAC-3'		
11	5'-GTGTTGACCTAATCATC-3'	48°C	Exon9
	5'-CTGCAAATAGCTTATA-3'		

*Annealing temperature.

PCR products were sequenced with the BigDye® Terminator sequencing kit version 1.1 on the 377 ABI PRISM® Sequencer Analyzer (Applied Biosystems).

We also analyzed urine samples from the proband and all family members for the presence of TMA and TMAO. A first urine sample was collected for 24 hours under normal dietary conditions (a diet not containing TMA-rich foods) and a second was collected for 6 to 8 hours after a 300g marine fish meal.

Urine samples were acidified to pH 3.0 with formic acid and stored at -20°C. Creatinine was measured by the Jaffe reaction on an autoanalyzer.

Derivatization of TMA was carried out according to the method by Johnson using ethyl bromoacetate as a derivative reagent [9,10]. Each sample was analyzed in duplicate.

Mutations analysis of nine exons of the *FMO3* gene was performed on all family members.

The proband was found heterozygous for the previously reported polymorphism c.472 G>A p. E158K (rs 2266782) in exon 4, and a G-to-A transition at codon 158 (GAG to AAG) resulting in a glutamic acid to lysine substitution (Glu158 to Lys158).

E158K polymorphism reduces *FMO3* catalytic activity that appears to vary depending on the substrate [11-13]. Previous *in vitro* expression studies showed that the K158 form of the protein is a poorer TMA *N*-oxygenator than the E158 form.

In some populations, this variant was found in a high degree of linkage disequilibrium with the E308G variant. When present on the same allele, the E158K and E308G exhibit an even more pronounced effect on *FMO3* function [11], even leading to mild or transient forms of TMAU [14].

The proband was heterozygous also for two polymorphisms in intronic regions: c.627+10 C>G (IVS5+10G>C) (rs 2066534) and c.485-21 G>A (IVS4-22G>A) (rs 1920149).

The first, a variant of uncertain functional relevance, was found in *cis* with E158K polymorphism [15] while the second was an intronic A-to-G substitution at the -21 position from the acceptor splice site of exon [6,16]. The parents and the eldest of two brothers were heterozygous for the same variants while the younger brother did not show any variation.

**Figure 1:**
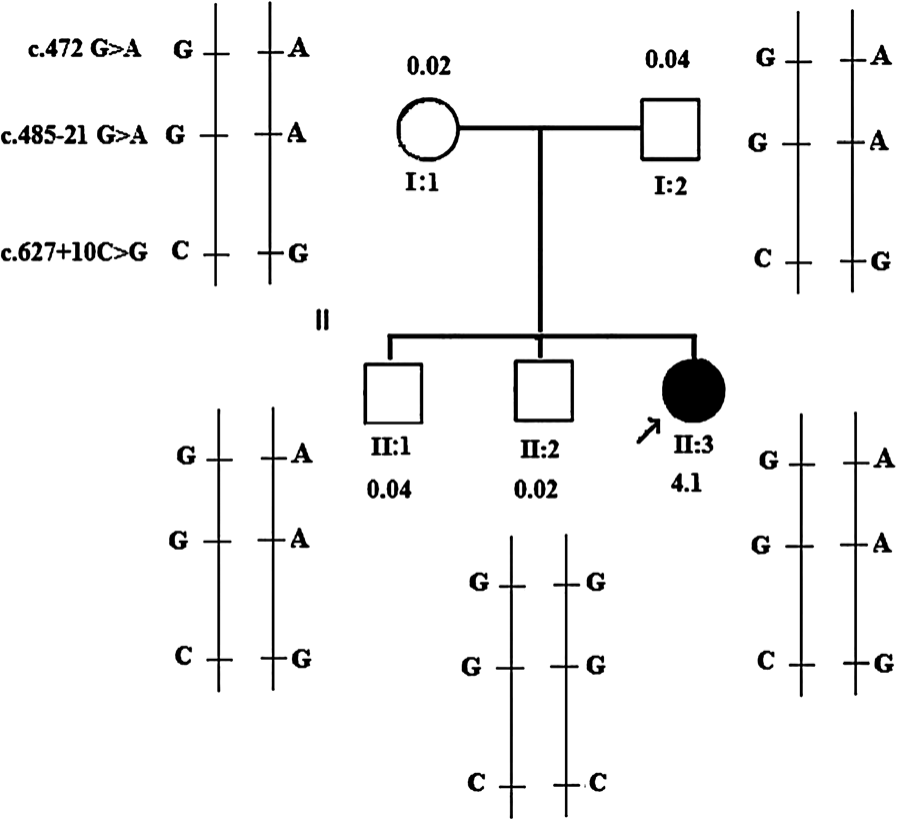
**Pedigree of the child with trimethylaminuria-like phenotype and flavin-containing monooxygenase 3 haplotype analysis of the child (arrow) and other family members.** The flavin-containing monooxygenase 3 complementary DNA sequence from GenBank accession number NM_006894.5 was used as a reference sequence where the A of the ATG translation initiation start site represents nucleotide +1. *N*-oxidation metabolic ratios for family members after a 300g marine fish meal.

Since the latter was wild-type it is possible to deduce that he has inherited the wild-type allele from each parent and that c.472 G>A, c.485-21 G>A and c.627+10C>G polymorphisms occurred in *cis* configuration on one of the two *FMO3* alleles of the father and mother.On the basis of this it is possible to infer that the proband and the elder of the two brothers, as well as the parents, were compound heterozygotes for the three polymorphisms (Figure 1). However, among them, only the proband showed a TMAU-like phenotype.

Therefore, we wanted to analyze the upstream region of *FMO3* gene in order to identify polymorphic variants that could affect the enzymatic activity. No variants were identified in any family members. Analysis of the urine samples collected for 24 hours from both parents and two brothers under normal dietary conditions (a diet not containing TMA-rich foods such as fish or eggs) showed the presence of relatively small amounts of TMA excreted. The *N*-oxidation metabolic ratio (TMA/TMAO ratio) for the four subjects ranged from 0.02 to 0.04.

For the proband the TMA excretion accounted for only 29.0% of total TMA excretion. The *N*-oxidation metabolic ratio for the proband was 2.4, two orders of magnitude greater than those observed for the parents and two brothers (affected ratio TMA/TMAO >0.2).

After oral TMA challenge, the amount of urinary TMA excreted as TMAO, in the parents and two brothers remained high and it was very similar to the values under normal dietary conditions. *N*-oxidation metabolic ratio ranged from 0.02 to 0.04.

After oral TMA challenge, in the proband, TMAO excretion accounted for 19.4% of total TMA excreted (Table 2).

**Table 2:** **Urinary excretion of trimethylamine and trimethylamine *N*-oxide by the proband and family members under normal dietary conditions and after 300g marine fish meal**

	**Normal dietary conditions**				**300g marine fish meal**			
	**TMA (mmol/mol Crn)**	**TMAO (mmol/mol Crn)**	**TMA/TMAO ratio**	**Amount of total TMA excreted as TMAO (%)**	**TMA (mmol/mol Crn)**	**TMAO (mmol/mol Crn)**	**TMA/TMAO ratio**	**Amount of total TMA excreted as TMAO (%)**
Mother	104	5960	0.02	98.3	480	19140	0.02	97
Father	70	1820	0.04	96.3	640	14000	0.04	95.6
Proband	117	48	2.4	29.0	738	178	4.1	19.4
Brother1	20	600	0.03	97.0	225	5420	0.04	99.0
Brother2	35	910	0.04	96	320	12220	0.02	97.0

Normal ratio: TMA/TMAO = 0.0025 to 0.055 (>85% TMAO).

Affected ratio: TMA/TMAO >0.2 (<20% TMAO).

*Abbreviations*: *Crn* creatine, *TMA* trimethylamine, *TMAO* trimethylamine *N*-oxide.

## Discussion

In this study we evaluated a 7-year-old girl with a clinical suspicion of TMAU. Results of molecular genetic studies revealed that she was heterozygous for three polymorphic variants one of which, the c.472 G>A, is known to cause a reduction in *FMO3* enzymatic activity. Among the other two variants, both intronic, the c. 485–21 G>A falls within the intron 4 splice acceptor site. The variant results in an increase in information content (R_i_) suggesting that it strengthens the respective splice site and potentially affects the splicing [17]. Both the parents and one of the two brothers had the same genotypic condition. However, in contrast to her parents and brother only the proband displays symptoms of TMAU.

This phenotypic manifestation was supported by data on urinary determination of TMA and TMAO that show an *N*-oxidation metabolic ratio for the proband equal to 2.4, two orders of magnitude greater than those observed for the parents and two brothers.

In addition, no variants in the upstream region of *FMO3* gene have been identified in the proband, absent in other family members, such as to justify this significant reduction in enzyme activity.

## Conclusions

The most likely explanation is that the proband has transient childhood TMAU [18].

In fact, *FMO3* and *FMO1* are subject to developmental and tissue-specific regulation and, in the liver, there is a developmental switch in the expression of these genes: *FMO1* functional activity decreases in early childhood time periods and a concomitant increase in functional activity of *FMO3* emerges [19].

*FMO3* expression is detectable in most individuals by 1 to 2 years of age and it is expressed at intermediate levels until approximately 11 years [2].

In the proband, a combination of one non-functional *FMO3* allele and immature expression of the other, functional, *FMO3* allele, may be sufficient to cause symptoms of the disorder. As the proband develops, the expression of her functional allele will increase and, consequently, her symptoms should eventually disappear. After all this is verifiable in the 16-year-old elder brother of the proband: he has the same genotype as the proband and parents but no TMAU symptoms. In this case, genetic factor(s) may have less impact on this phenotype. The large majority of cases with primary *FMO3* deficiency will present in early childhood and accurate diagnosis is essential for appropriate genetic counselling and their long-term management. Clinically, whether in the child or adult, TMAU cannot be considered a benign or ‘social’ condition, early diagnosis is important in children with TMAU so that appropriate dietary therapy may be introduced as soon as possible. Although initial indications of the disorder may be obtained by analysis of a single urine sample, this is not always reliable, especially when the child is ingesting a diet low in TMA precursors.

## Consent

Written informed consent was obtained from the patient’s parents for publication of this case report. A copy of the written consent is available for review by the Editor-in-Chief of this journal.

## Competing interests

The authors declare that they have no competing interests and that no financial support was obtained for the publication of this manuscript.

## Authors’ contributions

RD: design and conceptualization of the study, revising the manuscript. CS: carried out the molecular genetic studies. TE: TMA/TMAO urinary determination and data analysis. DB: provided clinical information. CR: design and conceptualization of the study, drafting of manuscript. AR: provided acquisition of data and sequence alignment*.* AS: design and conceptualization of the study, revising the manuscript. All authors read and approved the final manuscript.
